# Structural and Biochemical Characterization of the Early and Late Enzymes in the Lignin β-Aryl Ether Cleavage Pathway from *Sphingobium* sp. SYK-6*[Fn FN2]

**DOI:** 10.1074/jbc.M115.700427

**Published:** 2016-03-03

**Authors:** Jose Henrique Pereira, Richard A. Heins, Daniel L. Gall, Ryan P. McAndrew, Kai Deng, Keefe C. Holland, Timothy J. Donohue, Daniel R. Noguera, Blake A. Simmons, Kenneth L. Sale, John Ralph, Paul D. Adams

**Affiliations:** From the ‡ Joint BioEnergy Institute, Emeryville, California 94608,; the § Molecular Biophysics and Integrated Bioimaging Division, Lawrence Berkeley National Laboratory, Berkeley, California 94720,; the ¶ Biological and Engineering Sciences Center, Sandia National Laboratories, Livermore, California 94551,; the ‖ United States Department of Energy Great Lakes Bioenergy Research Center, Wisconsin Energy Institute, University of Wisconsin, Madison, Wisconsin 53726,; the Departments of ** Civil and Environmental Engineering and; ‡‡ Biochemistry, University of Wisconsin, Madison, Wisconsin 53706, and; the §§ Department of Bioengineering, University of California, Berkeley, California 94720

**Keywords:** biodegradation, biofuel, crystal structure, enzyme kinetics, lignin degradation

## Abstract

There has been great progress in the development of technology for the conversion of lignocellulosic biomass to sugars and subsequent fermentation to fuels. However, plant lignin remains an untapped source of materials for production of fuels or high value chemicals. Biological cleavage of lignin has been well characterized in fungi, in which enzymes that create free radical intermediates are used to degrade this material. In contrast, a catabolic pathway for the stereospecific cleavage of β-aryl ether units that are found in lignin has been identified in *Sphingobium* sp. SYK-6 bacteria. β-Aryl ether units are typically abundant in lignin, corresponding to 50–70% of all of the intermonomer linkages. Consequently, a comprehensive understanding of enzymatic β-aryl ether (β-ether) cleavage is important for future efforts to biologically process lignin and its breakdown products. The crystal structures and biochemical characterization of the NAD-dependent dehydrogenases (LigD, LigO, and LigL) and the glutathione-dependent lyase LigG provide new insights into the early and late enzymes in the β-ether degradation pathway. We present detailed information on the cofactor and substrate binding sites and on the catalytic mechanisms of these enzymes, comparing them with other known members of their respective families. Information on the Lig enzymes provides new insight into their catalysis mechanisms and can inform future strategies for using aromatic oligomers derived from plant lignin as a source of valuable aromatic compounds for biofuels and other bioproducts.

## Introduction

The production of renewable chemicals and advanced biofuels from lignocellulosic biomass is a potentially sustainable route to support the growing demand for energy and materials that are currently obtained from fossil fuels (1). Lignin is the primary obstacle to the efficient breakdown of lignocellulosic biomass, and there has therefore been considerable interest in processes for lignin degradation (2, 3). Lignin can also represent a major fraction of the plant cell wall carbon, so it has been estimated that the conversion of lignin into useful products could significantly improve the overall economics of lignocellulosic processing (2, 3). Biological, chemical, and hybrid routes for the conversion of lignin breakdown products are therefore of high interest.

Known pathways for fungal lignin degradation use chemically promiscuous enzymes that often depend on free radical intermediates to degrade this recalcitrant polymer (2, 3). Enzymatic pathways that catalyze the cleavage of specific bonds between the lignin building blocks have been less well characterized, with the exception of the Lig pathway from the bacterium *Sphingobium* sp. strain SYK-6 (4, 5). This organism and related bacteria have the ability to grow on a wide variety of dimeric aromatic compounds that can be derived from plant lignin (4–6). A cellular pathway has been characterized that performs β-aryl ether (β-ether)^3^ bond cleavage using three sequential Lig enzymes. These β-ether linkages account for 50–70% of interaromatic linkages in lignin (7), making β-ether cleavage an important target for lignin processing.

**FIGURE 1.**
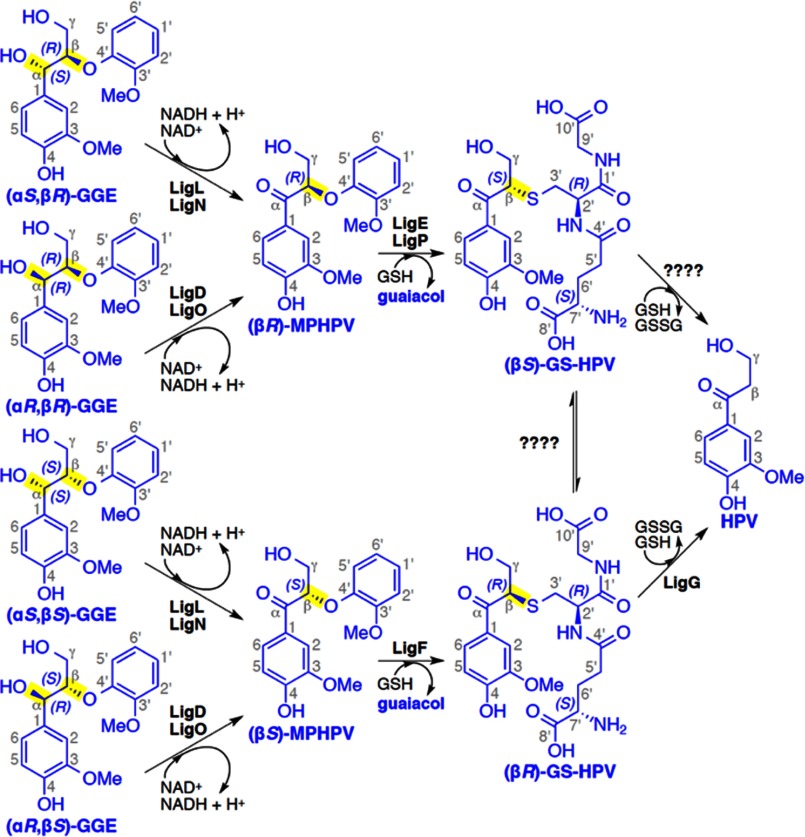
**The *Sphingobium* sp. strain SYK-6 β-etherase pathway.** Chiral carbons at which stereospecific reactions occur are highlighted (*yellow*). Stereospecific reactions for (α*S*,β*R*)-GGE and (α*S*,β*S*)-GGE oxidation (by LigL and LigN), (α*R*,β*R*)-GGE and (α*R*,β*S*)-GGE oxidation (by LigD and LigO), the GSH-dependent stereospecific cleavage reactions of (β*R*)-MPHPV (by LigE and LigP) and (β*S*)-MPHPV (by LigF), and the stereospecific lyase reaction of LigG with (β*R*)-GS-HPV are shown.

Using a model substrate, guaiacylglycerol-β-guaiacyl ether (GGE; see Fig. 1), enzymes that catalyze the three reactions in the β-ether cleavage pathway have been identified (4, 8, 9). Each of the Lig systems characterized to date uses a set of enzymes that are each specific for different stereoisomers in the substrates (4, 8, 9). The lignin polymer is assembled via combinatorial radical-mediated chemical coupling reactions in plant cells, and it appears that the Lig enzymes have evolved to accommodate the different chiral centers that are present in the plant-derived racemic materials (8).

The first step in the Lig pathway is a stereospecific oxidation of the benzylic alcohol in the GGE substrate to produce β-(2-methoxyphenoxy)-γ-hydroxypropiovanillone (MPHPV), which is catalyzed by nicotinamide adenine dinucleotide (NAD^+^)-dependent Cα-dehydrogenases LigD, LigL, LigN, and LigO. The LigD and LigO enzymes catalyze the oxidation of the (α*R*)-substrates (α*R*,β*R*)-GGE and (α*R*,β*S*)-GGE, whereas LigL and LigN are specific for the α(*S*)-configured substrates (α*S*,β*R*)-GGE and (α*S*,β*S*)-GGE. The next reaction is catalyzed by the enzymes LigE/LigP and LigF, which are members of the glutathione *S*-transferase (GST) superfamily. These stereospecifically catalyze the glutathione (GSH)-dependent cleavage of the β-ether linkage in MPHPV to generate β-glutathionyl-γ-hydroxypropiovanillone (GS-HPV) and guaiacol. The final step consists of a GSH-dependent lyase, LigG, that catalyzes the elimination of the GSH thioether linkage in (β*R*)-GS-HPV to produce glutathione disulfide (GSSG) and γ-hydroxypropiovanillone (HPV). It is the latter that is used by *Sphingobium* sp. strain SYK-6 for growth (5, 9). It is currently unknown whether the (β*S*)-GS-HPV stereoisomer is converted to HPV by an as yet unidentified stereospecific enzyme related to LigG or whether it racemizes to (β*R*)-GS-HPV for conversion by LigG. Although most of the reported studies have monitored activity of these enzymes with model aromatic substrates, there are reports of Lig pathway-mediated release of aromatics from plant-derived lignin fractions *in vitro* (10, 11). When taken together, the properties of Lig enzymes are suited to the cleavage of small aromatic oligomers that could be derived from lignin.

In the work presented here, we biochemically and structurally characterize native and mutant enzymes involved in the early (Cα-dehydrogenase) and late (glutathione lyase) steps of the Lig-dependent β-ether degradation pathway. The crystal structures of the Cα-dehydrogenases LigD, LigL, and LigO were solved as apoenzymes, with cosubstrate NADH (the reduced form of NAD^+^) bound, or in the ternary complex of protein-NADH-GGE, providing a complete structural picture of each of the predicted enzymatic states in the catalytic cycle. Insight into the last reaction of the Lig pathway is provided by the crystal structures of apo-LigG and the LigG·GS-AV (β-glutathionyl-acetoveratrone) substrate analog complex (supplemental Fig. 1). These results add considerably to our mechanistic understanding of a bacterial β-ether degradation pathway that in the future could play a key role in the conversion of lignin breakdown products into fuels and chemicals.

## Experimental Procedures

### Cloning, Expression, and Purification of LigD, LigO, LigL, and LigG

#### 

##### Gene Cloning

LigD, LigO, LigL, and LigG were synthesized and cloned into a custom vector (pCPD) assembled by Genscript (Piscataway, NJ). This vector combined the pVP16 backbone (provided by the Center for Eukaryotic Structural Genomics, Madison, WI) with the gene of interest and a C-terminal fusion protein tag composed of the *Vibro cholerae* MARTX toxin cysteine protease domain (CPD). During protein purification, the CPD tag can be activated by the addition of inositol hexakisphosphate, cleaving at a leucine positioned between the protein of interest and CPD. Descriptions of the gene constructs for LigD, LigO, LigL, and LigG are summarized in supplemental Fig. 2.

##### Enzyme Purification

NEB Express protein expression cells (New England Biolabs, Inc., Ipswich, MA) containing either pCPD-LigD, pCPD-LigO, or pCPD-LigL were expressed in 500-ml Terrific broth cultures in 2-liter non-baffled flasks using 1:100 ratios with the overnight cultures. The cultures were grown at 37 °C with swirling at 200 rpm until the *A*_600_ reached ∼1.2. The temperature was lowered to 20 °C, and isopropyl 1-thio-β-d-galactopyranoside was added to a 1.0 mm final concentration for the induction of LigD, LigO, and LigL expression. After 24 h, the cells were harvested by centrifugation at 5000 × *g* for 20 min. pCPD-LigG was expressed in *Escherichia coli* B834 (Novagen) using autoinducing selenomethionine medium as described previously by Sreenath *et al.* (12). Briefly, 1–3 colonies were picked and transferred to 100 ml of PA-0.5G culture at 25 °C and swirled at 300 rpm overnight. A culture aliquot (20 ml) was then used to inoculate 2-liter bottles containing 480 ml of PASM-5052 medium incubated at 25 °C with shaking at 250 rpm for 24 h. The cells containing selenomethionine-labeled LigG were harvested by centrifugation at 5000 × *g* for 20 min. Harvested cells were resuspended in 30 ml of lysis buffer (50 mm HEPES buffer, pH 7.4, 150 mm NaCl, and 40 mm imidazole) and lysed by an Avestin EmulsiFlex-C3 homogenizer. The C-terminally His-tagged proteins were purified from the clarified supernatant using precharged nickel-immobilized metal ion affinity chromatography resin (GE Healthcare). After protein binding and washing twice with lysis buffer, inositol hexakisphosphate was added to a final concentration of 200 μm. Note that the inositol hexakisphosphate was first diluted to 10 mm in lysis buffer to neutralize the acidic pH of the stock solution. After 1 h of incubation, the resin was washed with 1 ml of lysis buffer to elute the cleaved protein. Following buffer exchange into 20 mm Tris, pH 8, proteins were further purified using a HiTrap Q HP anion exchange column. Fractions containing the protein of interest, as confirmed by SDS-PAGE, were pooled and concentrated. Final protein clean up was performed using gel filtration on a Superdex 200 10/300 GL column (GE Healthcare); >90% purity was observed on SDS-polyacrylamide gels for LigD, LigO, LigL, and LigG samples used for enzyme kinetic and crystallographic assays.

### Enzyme Kinetic Assays

#### 

##### NAD^+^-dependent Dehydrogenation Assays

*In vitro* NAD-dependent dehydrogenase assays with LigL were performed in an aqueous assay buffer with 25 mm phosphate (pH 6.5–7.5) or Tris (pH 7.5–9) and 5 mm NAD at 30 °C with substrate concentrations ranging from 6.25 to 100 μm and an enzyme concentration of 3.125 nm. Enantiopure preparations of (α*S*,β*S*)-GGE and (α*S*,β*R*)-GGE were synthesized as described previously (13). After incubating the enzyme with substrate for 10 min, the reaction was quenched by the addition of an equivalent volume of 5% (v/v) formic acid in water. Each sample was then subjected to reverse-phase HPLC using a Kinetex^TM^ 5-μm phenyl-hexyl 100-Å, LC column, 250 × 4.6 mm, attached to an Agilent 1100 high pressure liquid chromatograph. Samples and external standards were quantified by UV absorption at 280 and 300 nm. The HPLC mobile phase was a mixture of aqueous (0.1% (v/v) formic acid in water) and acetonitrile (0.1% (v/v) formic acid) at a flow rate of 1 ml/min. MPHPV and GGE concentrations were quantified in order to calculate the specific activity of each reaction. The average of the triplicate assays was reported.

##### GSH-dependent Glutathione Lyase Assays

*In vitro* GSH-dependent glutathione lyase assays with LigG were performed in an aqueous assay buffer with 25 mm Tris, pH 9, and 5 mm GSH at 30 °C with substrate concentrations ranging from 3.125 to 50 μm and an enzyme concentration of 1 nm. The GS-AV substrate was synthesized as described previously (8). An enantiopure preparation of (β*R*)-GS-HPV was synthesized by reacting a known quantity of enantiopure (β*S*)-MPHPV with LigF until the reaction reached completion (verified by the disappearance of (β*S*)-MPHPV via HPLC), after which LigF was heat-inactivated (verified by the absence of catalytic activity upon the addition of fresh (β*S*)-MPHPV). After incubating the enzyme with substrate for 10 min, the reaction was quenched by the addition of an equivalent volume of 5% (v/v) formic acid in water. Samples were then measured by HPLC as described above.

### Crystallization of LigD, LigO, LigL, and LigG

LigD, LigO, LigL, and LigG were dialyzed against 20 mm HEPES, pH 7.4, and 50 mm NaCl and concentrated to 10–15 mg ml^−1^ prior to crystallization trials. The optimal conditions for crystallization of the proteins were as follows: LigD, 0.1 m Hepes, pH 7.5, and 1.5 m lithium sulfate; LigO, 0.1 m ammonium citrate, 0.1 m MES, pH 5.5, 20% (w/v) PEG 3,350, and 5% (v/v) isopropyl alcohol; LigL, 0.2 m magnesium chloride, 0.1 m Hepes, pH 7.5, and 25% PEG 3,350; and LigG, 0.1 m Bistris propane, pH 7.0, and 1.5 m ammonium sulfate. LigO, LigL, and LigG crystals were obtained after 1–7 days, whereas the LigD crystals were obtained after 45 days. All of the crystals were obtained by the sitting drop vapor diffusion method with the drops consisting of a mixture of 0.2 μl of protein solution and 0.2 μl of reservoir solution. The crystallization experiments were performed at 295 K.

### X-ray Data Collection and Structure Determination

The crystals of LigD, LigO, LigL, and LigG were placed in a reservoir solution containing 10–20% (v/v) glycerol and then flash-cooled in liquid nitrogen. The x-ray data sets for LigD, LigO, LigL, and LigG were collected at the Berkeley Center for Structural Biology beamlines 8.2.1 and 8.2.2 of the Advanced Light Source at Lawrence Berkeley National Laboratory. Data sets were indexed and scaled using HKL2000 (14). LigD, LigO, and LigL crystal structures were determined by the molecular replacement method with the program *PHASER* (15) within the *Phenix* suite (16). LigD was solved using as a search model the Protein Data Bank coordinates of 3IOY, which has 35% sequence identity with the target. The LigO and LigL crystal structures were solved using the LigD coordinates as the search model. The results from the molecular replacement for LigD, LigO, and LigL showed a translation function Z-score of 15.1, 20.7, and 21.2, respectively, where translation function Z-score values of >8 strongly suggest correct solutions (16). The atomic positions obtained from molecular replacement and the resulting electron density maps were used to build the LigD, LigO, and LigL structures and initiate crystallographic refinement and model rebuilding. The crystal structure of LigG was solved using selenomethionine-labeled protein by single-wavelength anomalous dispersion methods (17) with the *phenix.autosol* (18) and *phenix.autobuild* (19) programs. The figure of merit from single-wavelength anomalous dispersion phasing was 0.54, indicating a good phase quality (16). Structural refinement was performed using the *phenix.refine* program (20). Manual rebuilding using *COOT* (21) and the addition of water molecules allowed construction of the final models. Root mean square deviation differences from ideal geometries for bond lengths, angles, and dihedrals were calculated with *Phenix* (16). The overall stereochemical quality of all final models was assessed using the program *MOLPROBITY* (22). Superposition of the models for structural comparison was performed using *COOT* (21).

## Results

### NAD^+^-dependent Cα-dehydrogenases (LigD, LigO, and LigL)

#### 

##### Cα-dehydrogenase Structural Analysis

We have solved the crystal structures of LigD, LigO, and LigL, which by amino acid sequence alignment belong to the short chain dehydrogenase/reductase (SDR) superfamily of enzymes (23). Each of these Lig enzymes catalyzes the oxidation of the benzylic alcohol of GGE to form MPHPV (Fig. 1). Data collection, refinement, and model statistics for LigD, LigO, and LigL structures are summarized in Table 1.

**TABLE 1:** **Summary of crystal parameters, data collection, and refinement statistics** Values in parentheses are for the highest resolution shell.

	LigD-apo	LigD·NADH	LigO-apo	LigO·NADH	LigL-apo	LigL·NADH	LigL·NADH·GGE	LigG-apo	LigG·GS-AV
**Crystal parameters**									
Space group	P3_1_21	P3_1_21	P3_2_21	P3_2_21	C2	C2	C2	P3_2_21	P3_2_21
Unit-cell parameters (Å)	*a* = *b* = 61.30, *c* = 134.66	*a* = *b* = 61.76, *c* = 134.71	*a* = *b* = 79.23, *c* = 72.18	*a* = *b* = 79.29, *c* = 71.97	*a* = 126.64, *b* = 74.93, *c* = 56.28, β = 104.53	*a* = 126.04, *b* = 72.81, *c* = 58.97, β = 105.36	*a* = 125.71, *b* = 72.71, *c* = 58.87, β = 105.17	*a* = *b* = 64.66, *c* = 120.73	*a* = *b* = 61.30, *c* = 134.66

**Data collection statistics**									
Wavelength (Å)	1.000	1.000	1.000	0.977	1.000	1.000	0.977	1.000	0.977
Resolution range (Å)	50–2.64 (2.69–2.64)	50–2.01 (2.04–2.01)	50–1.80 (1.83–1.80)	50–1.70 (1.73–1.70)	50–1.60 (1.63–1.60)	50–1.50 (1.53–1.50)	50–1.60 (1.63–1.60)	50–1.11 (1.13–1.11)	50–1.40 (1.42–1.40)
No. of reflections (measured/unique)	98,324/9044	20,9410/19,774	268,555/24,752	315,672/29,261	248,968/66,935	304,447/82,006	252,858/67,529	1,048,340/115,374	626,571/58,125
Completeness (%)	99.9 (99.5)	96.5 (76.3)	100 (100)	100 (99.5)	100 (99.9)	100 (100)	100 (100)	99.8 (99.0)	100 (100)
*R*_merge_*^a^*	0.089 (0.49)	0.081 (0.47)	0.049 (0.78)	0.082 (0.92)	0.070 (0.60)	0.057 (0.61)	0.048 (0.56)	0.060 (0.46)	0.079 (0.83)
Redundancy	10.9 (7.4)	10.6 (6.7)	10.8 (10.9)	10.8 (9.0)	3.7 (3.5)	3.7 (3.5)	3.7 (3.7)	9.1 (5.3)	10.8 (10.8)
Mean *I*/σ(*I*)	25.2 (2.80)	26.1 (2.10)	46.3 (1.95)	28.1 (1.79)	17.29 (1.57)	21.6 (1.70)	29.8 (1.93)	24.0 (2.6)	23.8 (2.2)

**Refinement and model statistics**									
Resolution range (Å)	50–2.64 (3.03–2.64)	50–2.01 (2.07–2.01)	50–1.80 (1.84–1.80)	34–1.70 (1.74–1.70)	47–1.60 (1.64–1.60)	48–1.50 (1.51–1.50)	48–1.60 (1.64–1.60)	41–1.11 (1.12–1.11)	41–1.40 (1.43–1.40)
No. of reflections (work/test)	8829/417	16,006/1620	23,770 (1934)	29,201 (1999)	66,929 (2000)	81,999 (4111)	67,525/1999	115,285/5784	58,064/2003
*R*_cryst_*^b^*	0.211 (0.328)	0.210 (0.374)	0.177 (0.251)	0.174 (0.259)	0.149 (0.220)	0.132 (0.226)	0.139 (0.220)	0.167 (0.266)	0.139 (0.185)
*R*_free_*^c^*	0.254 (0.399)	0.248 (0.377)	0.206 (0.309)	0.199 (0.310)	0.165 (0.233)	0.154 (0.238)	0.161 (0.237)	0.182 (0.272)	0.164 (0.240)
Root mean square deviation									
Bonds (Å)	0.007	0.008	0.003	0.004	0.006	0.009	0.006	0.019	0.013
Angles (degrees)	0.939	1.003	0.773	0.900	1.051	1.313	1.122	1.837	1.558
All-atom Clashscore	1.1	1.4	0.7	1.6	3.7	4.7	0.8	8.5	7.0
*B* factor (protein/solvent) (Å^2^)	99.8/NA*^d^*	58.9/55.75	44.39/44.93	46.5/32.4	21.13/32.66	18.06/31.56	27.35/36.24	13.94/27.13	20.72/32.57
No. of protein atoms	1724	1724	2218	2234	4061	4851	4813	2636	2165
No. of waters	0	12	142	131	541	649	559	441	334
No. of auxiliary molecules	0	1 NADH	0	1 NADH	0	2 NADH	2 NADH, 1 GGE, 1 Mg	1 SO_4_	1 GS-AV, 1 SO_4_

**Ramachandran plot (%)**									
Favorable region	97.9	97.8	97	96.3	98.3	98.2	98.0	98.1	97.7
Additional allowed region	2.1	2.2	3.0	3.7	1.7	1.8	2.0	1.9	2.3
Disallowed region	0	0	0	0	0	0	0	0	0
Protein Data Bank code	4Y98	4Y9D	4YA6	4YAC	4YAE	4YAG	4YAI	4YAP	4YAV

*^a^ R*_merge_ = Σ_h_ Σ_i_ I_i_ (h)–<I(h)> /Σ_h_Σ_i_ I_i_(h), where I_i_(h) is the intensity of an individual measurement of the reflection and <I(h)> is the mean intensity of the reflection.

*^b^ R*_cryst_ = Σ_h_ ‖F_obs_ – F_calc_‖/Σ_h_ F_obs_ , where F_obs_ and F_calc_ are the observed and calculated structure-factor amplitudes, respectively.

*^c^ R*_free_ was calculated as *R*_cryst_ using 5.0% of randomly selected unique reflections that were omitted from the structure refinement.

*^d^* Not applicable.

**FIGURE 2.**
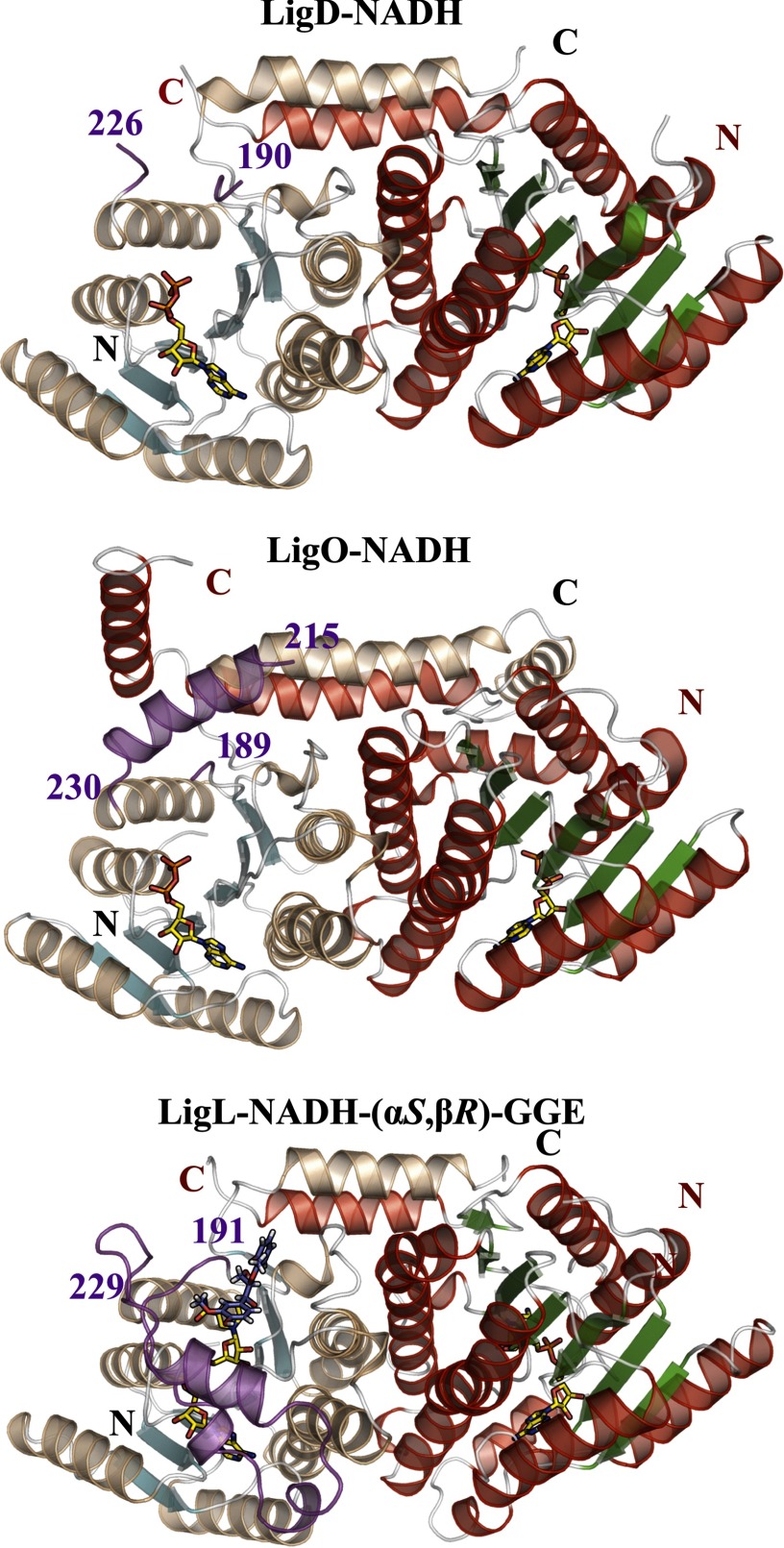
**Schematic representations of the biological dimers of LigD·NADH, LigO·NADH, and LigL·NADH·(α*S*,β*R*)-GGE, showing the overall SDR family fold composed of a central Rossmann fold.** The most sequence-divergent region of SDR family members is the substrate binding loop represented in *magenta*. This region is disordered in all of the crystal structures solved in the apo-forms (LigD, LigO, and LigL) and in the structures of the LigD·NADH and LigO·NADH complexes. The apo-LigO and LigO·NADH structures revealed a partially ordered region with an α-helix at the N terminus of the substrate binding loop. This loop is ordered and modeled in the binary complex of LigL·NADH and ternary complex structure of LigL·NADH·(α*S*,β*R*)-GGE, indicating a conformational change of this loop upon cosubstrate binding.

**FIGURE 3.**
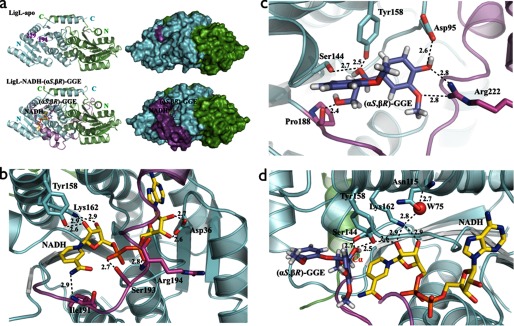
*a*, schematic and molecular surface representations of apo-LigL and the LigL·NADH·(α*S*,β*R*)-GGE complex. The substrate binding loop (residues 191–229) is completely disordered in the apo-LigL structure. In the LigL·NADH·(α*S*,β*R*)-GGE complex structure the substrate binding loop region (*magenta*) acts as a lid above the NADH and GGE binding sites. *b*, the active site of LigL in complex with NADH showing the interactions involving the co-substrate NADH. Residue Asp^36^ contacts the 2′- and 3′-hydroxyl groups of the adenosine ribose sugar, the catalytic residues Tyr^158^ and Lys^162^ contact the nicotinamide ribose sugar, the nicotinamide moiety interacts with the main chain nitrogen atom from Ile^191^, and the phosphate groups interact with side chain atoms from Ser^193^ and the main chain nitrogen atom from Arg^194^. *c*, the substrate binding site for LigL·NADH·(α*S*,β*R*)-GGE showing residues Asp^95^, Ser^144^, Tyr^158^, Pro^188^, and Arg^222^ that interact directly with the GGE substrate. *d*, active site of LigL·NADH·(α*S*,β*R*)-GGE showing the catalytic tetrad N^115^-S^144^-Y^158^-K^162^ and a water molecule (*W75*) involved in the extended proton relay system described for the SDR family (24). *Broken lines* represent hydrogen bonds, and distances are shown in Å.

The SDR family is characterized as a large group of NAD(P)H (2′-phosphorylated NADH)-dependent enzymes displaying an α/β folding pattern containing a Rossman fold (24); this overall organization is seen in the LigD, LigO, and LigL crystal structures (Fig. 2). LigD, LigO, and LigL are classified as classical SDR members that have a core structure of ∼300 residues and share the ^11^TG*XXX*G*X*(G/A)^18^ sequence motif at the cofactor binding site and the catalytic tetrad N^115^-S^144^-Y^158^-K^162^ (LigL numbering) (25). The superposition of the LigD, LigO, and LigL structures using Cα atoms shows a root mean square deviation for LigD-LigO of 0.88 Å, for LigD-LigL of 0.70 Å, and for LigO-LigL of 0.93 Å, indicating that the enzymes display similar overall structural features. A sequence alignment of LigD, LigO, and LigL shows an identity between LigD-LigO of 40%, LigD-LigL of 38%, and LigO-LigL of 37%. In these alignments, the most sequence-divergent region is in the predicted substrate binding loop (supplemental Fig. 3). This proposed substrate binding region is disordered in all of the crystal structures solved in the apo-form (LigD, LigO, and LigL) and in the structures of the LigD·NADH and LigO·NADH complexes. However, in the structures of the binary complex of LigL·NADH and the ternary complex structure of LigL·NADH·(α*S*,β*R*)-GGE, this region is well ordered, suggesting that this is a flexible loop that undergoes a major conformational change upon cosubstrate binding to the enzyme (Fig. 3*a*).

Despite the high quality of the diffraction data for LigD·NADH (2.0 Å resolution) and LigO·NADH (1.7 Å resolution), disorder was observed for the nicotinamide moiety of NADH, and only the adenosine diphosphate of the cosubstrate was visible in the electron density maps (supplemental Fig. 4*a*). Similar results were observed in crystals of several other SDR members, including a stereospecific short chain alcohol dehydrogenase solved at 1.0 Å resolution, suggesting that this is an intrinsic feature related to the flexibility of the nicotinamide region for some members of this family (26). However, the ternary complex structure of LigL·NADH·(α*S*,β*R*)-GGE reveals clear electron density for both NADH and the (α*S*,β*R*)-GGE substrate (supplemental Fig. 4*b*). In this case, LigL interacts with the adenosine region of NADH via residues located in the loop between strand β2 and helix α2; Asp^36^ contacts the 2′- and 3′- hydroxyl groups of the adenosine ribose sugar, the catalytic residues Tyr^158^ and Lys^162^ contact the nicotinamide ribose sugar, the nicotinamide moiety interacts with the main chain nitrogen atom from Ile^191^, and the phosphate groups interact with side chain atoms from Ser^193^ and the main chain nitrogen atom of Arg^194^ (Fig. 3*b*). These LigL residues are located at the start of the observed substrate binding loop, indicating that the hydrogen bonds between residues Ile^191^, Ser^193^, and Arg^194^ and the NADH are important in stabilizing the substrate binding loop “closed” conformation (Fig. 3*a*). The existence of a negatively charged aspartate (Asp^36^, Asp^37^, and Asp^38^ in LigL, LigD, and LigO, respectively) that interacts with the hydroxyl groups of the adenosine ribose is probably responsible for favoring NADH rather than NADPH as the cosubstrate. The binding of NAD(P)H is probably unfavorable due to electrostatic repulsion between the aspartate residue and the NADPH 2′-phosphate group (26–28).

The structure of the LigL·NADH·(α*S*,β*R*)-GGE ternary complex reveals the interactions between the LigL substrate binding site residues and the (α*S*,β*R*)-GGE substrate. The (α*S*,β*R*)-GGE substrate makes direct interactions, via hydrogen bonds, with Asp^95^, Ser^144^, Tyr^158^, the main chain carbonyl group of Pro^188^, and Arg^222^ located at the C terminus of the substrate binding loop region (Fig. 3*c*). From the group of residues Asp^95^, Ser^144^, Tyr^158^, Pro^188^, and Arg^222^ of LigL identified as directly contacting the (α*S*,β*R*)-GGE substrate, the sequence alignment showed that the catalytic Ser^144^/Tyr^158^ and Pro^188^ are conserved in the LigD and LigO sequences (supplemental Fig. 3). A molecular model of LigL in complex with NADH and the other α(*S*)-configured substrate (α*S*,β*S*)-GGE was created in order to predict the interaction between the LigL and (α*S*,β*S*)-GGE. All of the interactions observed between LigL and (α*S*,β*R*)-GGE (Fig. 3*c*) are conserved in the LigL-(α*S*,β*S*)-GGE model. An extra hydrogen bond between Ser^144^ and the hydroxyl group attached to Cγ of (α*S*,β*S*)-GGE was observed in the model of LigL·NADH·(α*S*,β*S*)-GGE (supplemental Fig. 5). The catalytic “extended proton relay system” mechanism for this class of enzymes, as proposed by Filling *et al.* (29), describes the role of the N^115^-S^144^-Y^158^-K^162^ tetrad, the 2′-OH group of NAD^+^, and a water molecule (Wat^75^ in Fig. 3*d*) in the transfer of a proton from the active site to the bulk solvent. By analogy, the LigL tyrosine residue 158 (Tyr^158^) functions as the catalytic base, the serine (Ser^144^) stabilizes the substrate via a hydrogen bond to the Cα-OH group of (α*S*,β*R*)-GGE, and lysine (Lys^162^) interacts with the nicotinamide ribose sugar and is proposed to lower the p*K_a_* of the tyrosine. Finally, the asparagine residue (Asn^115^) stabilizes the water molecule involved in the extended proton relay system (29). The conversion from GGE to MPHPV during the Cα-dehydrogenase reaction is achieved by the loss of a proton and a hydride ion from the substrate, creating NADH from NAD^+^, and transfer of the proton to the bulk solvent (Fig. 1). The ternary complex LigL·NADH·(α*S*,β*R*)-GGE reveals that the hydrogen bond to the Cα position of (α*S*,β*R*)-GGE is directed toward the NADH cofactor, whereas the hydrogen atom from the Cα-OH group of (α*S*,β*R*)-GGE is stabilized by the catalytic Ser^144^ (Fig. 3*d*).

##### Cα-dehydrogenase Enzymatic Analysis

We observed that the dehydrogenation of GGE to MPHPV is governed by solution pH for all four Lig enzymes tested, with equilibrium conversion reaching ∼90% at pH 9 but only 25% at pH 7 (supplemental Fig. 6). This is not unexpected, given the contribution of hydrogen ions to this reaction. Enzyme kinetics for LigL were therefore conducted at pH 9 to reduce the influence of equilibrium effects on kinetic parameters. LigL exhibited Michaelis-Menten kinetics using both (α*S*,β*R*)-GGE and (α*S*,β*S*)-GGE stereoisomers (Table 2) with a higher turnover number (*k*_cat_) and a lower Michaelis constant (*K_m_*) toward the latter substrate. The extra hydrogen bond interaction observed in the LigL·NADH·(α*S*,β*S*)-GGE model could to be the reason for this higher turnover. As expected from a prior report, LigL did not show detectable dehydrogenation of (α*R*,β*S*)-GGE or (α*R*,β*R*)-GGE substrates (9).

**TABLE 2:** **Kinetic parameters, determined from Michaelis-Menten curves for NAD^+^-dependent Cα-dehydrogenase LigL and its variants with substrates (α*S*,β*R*)-GGE and (α*S*,β*S*)-GGE at pH 9.0**

Enzyme	Substrate	*V*_max_	Percentage of WT activity with (α*S*,β*R*)-GGE	*k*_cat_	*K_m_*	*k*_cat_/*K_m_*
		*units mg*^−*1*^	%	*s*^−*1*^	μm	*mm*^−*1*^ *s*^−*1*^
LigL	(α*S*,β*S*)-GGE	33.7 ± 2.9	154	7.5 ± 0.1	10.9 ± 0.8	688 ± 53
LigL	(α*S*,β*R*)-GGE	21.8 ± 2.1	100	4.9 ± 0.3	20.3 ± 3.2	240 ± 41
LigL-R222A	(α*S*,β*R*)-GGE	<0.01	<0.1			
LigL-D95A	(α*S*,β*R*)-GGE	52.7 ± 6.5	241			
LigL-P188A	(α*S*,β*R*)-GGE	36.3 ± 3.8	166			
LigL-P188A-R222A	(α*S*,β*R*)-GGE	<0.01	<0.1			

The crystal structure of the LigL·NADH·(α*S*,β*R*)-GGE complex revealed contacts between the substrate and Asp^95^, Ser^144^, Pro^188^, and Arg^222^ (Fig. 3*c*). Prior analysis of SDR enzymes predicts that residues analogous to Ser^144^ and Tyr^158^ are essential members of the catalytic tetrad N^115^-S^144^-Y^158^-K^162^. The remaining three residues (Asp^95^, Pro^188^, and Arg^222^) were therefore individually mutated to alanine to explore their contributions to enzyme catalysis using the same (α*S*,β*R*)-GGE stereoisomer (Table 2). A 2.5 times higher enzymatic activity was observed for the mutant LigL-D95A compared with its wild-type counterpart, indicating that Asp^95^ is not required for the binding of the (α*S*,β*R*)-GGE substrate. There are interactions between Pro^188^ and the (α*S*,β*R*)-GGE substrate that occur via a hydrogen bond to the main chain carbonyl group of Pro^188^. Although the mutant LigL-P188A does not eliminate this interaction, it could influence the substrate binding loop flexibility and consequently the enzyme activity. The LigL-P188A mutant showed an ∼60% increase in enzymatic activity compared with wild-type LigL. Finally, the LigL-R222A mutant protein lacked detectable enzyme activity, indicating that Arg^222^ is a key residue. Arg^222^ is also the only residue located in the substrate binding loop region that interacts directly with the (α*S*,β*R*)-GGE substrate. Sequence alignment of LigL, LigD, and LigO showed that an Arg at this position is not conserved in all three proteins (supplemental Fig. 3). Because LigL catalyzes the conversion of α(*S*)-configured substrates (α*S*,β*R*)-GGE and (α*S*,β*S*)-GGE, and LigD and LigO catalyze the conversion of the α(*R*)-substrates (α*R*,β*R*)-GGE and (α*R*,β*S*)-GGE, we hypothesize that the difference in the side chain at this position is related to substrate stereoisomer recognition by these enzymes.

### GSH-dependent Glutathione Lyase (LigG)

#### 

##### Lyase Structural Analysis

We obtained apo-LigG and LigG·GS-AV crystals that diffracted to 1.1 and 1.4 Å resolution, respectively. Data collection, refinement, and model statistics for LigG are summarized in Table 1. The crystal structure of LigG was solved using selenomethionine-labeled protein by single-wavelength anomalous dispersion methods (17). LigG belongs to the Omega class of GSTs that have a catalytic cysteine residue, as confirmed by loss of activity in the LigG-C15S variant (30). The LigG enzyme catalyzes cleavage of the (β*R*)-GS-HPV β-thioether bond. The LigG enzymatic mechanism consists of two reaction steps. Initially, Cys^15^ forms a disulfide bond with the sulfur atom of the glutathione moiety from the GS-HPV substrate, releasing the HPV portion. Then a second GSH molecule enters the active site, and sulfide exchange with Cys^15^-GS takes place, forming and releasing a GSSG molecule, restoring the enzyme to a substrate-accepting state (31, 32) (Fig. 1). The structure of the LigG·GS-AV complex supports a reaction mechanism in which a disulfide bond is formed between sulfur atoms of the GS-AV substrate analog and the catalytic residue Cys^15^.

**FIGURE 4.**
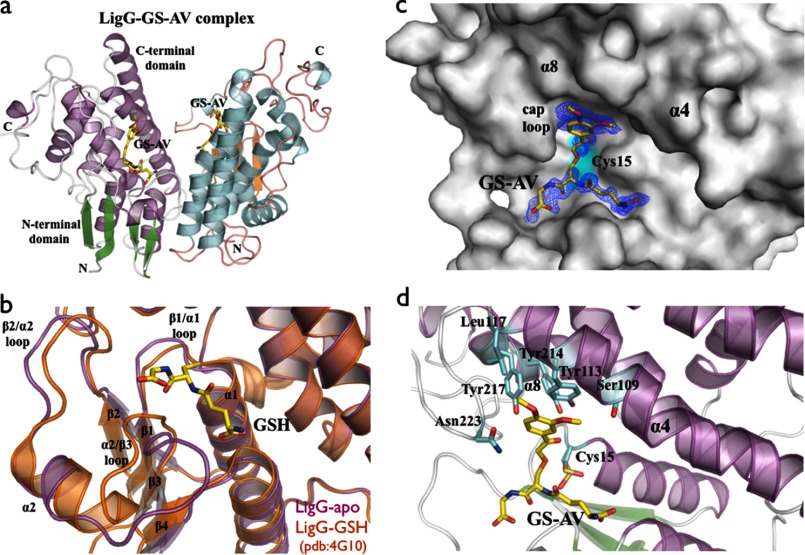
*a*, overall schematic representation of the LigG·GS-AV complex dimer. *b*, superposition of the GSH binding site of apo-LigG (*magenta*) and LigG-GSH (Protein Data Bank entry 4G10) (*orange*) structures (30). Significant conformational changes of the GSH binding site were observed on the loop regions at the N-terminal domain connecting the β1/α1, β2/α2, and α2/β3 structural elements. *c*, molecular surface representation of the LigG monomer in complex with the GS-AV substrate analog. A feature-enhanced map (35) contoured at 1.0 σ is shown in *blue* around the GS-AV substrate analog (this molecule was omitted from the model to reduce bias). The position of the catalytic Cys^15^ residue is highlighted in *cyan. d*, active site of the LigG·GS-AV complex. The glutathionyl moiety of the GS-AV substrate sits on the top of the four β-strands of the N-terminal thioredoxin domain. In addition, the AV moiety of the GS-AV molecule contacts the C-terminal α-helical domain of LigG via residues Ser^109^, Tyr^113^, and Leu^117^ on α4, residues Tyr^214^ and Tyr^217^ on α8, and Asn^223^ on the cap loop region composed of the residues ^220^GGGNG^224^.

The LigG structure possesses the canonical GST domain fold with an N-terminal thioredoxin domain (β1α1β2α2β3β4α3) and a C-terminal α-helical domain composed of six α-helices (Fig. 4*a*). A comparison between the apo-LigG form and that of a LigG-GSH complex (30) revealed a conformational change upon GSH binding at the site located in the loops between β1/α1, β2/α2, and α2/β3 of the N-terminal domain (Fig. 4*b*). In order to obtain the LigG structure in a complex with the substrate analog GS-AV (Fig. 4, *c* and *d*), ∼1-year-old crystals of apo-LigG were used for soaking experiments. These older crystals show oxidation of the catalytic Cys^15^ that rendered the enzyme inactive and oxidation of Cys^94^ but no oxidation of Cys^196^. Evidence for oxidation was the presence of additional m*F_o_* − D*F_c_* electron density in maps contoured at 3.0 σ around the sulfur atoms (supplemental Fig. 7). Similar oxidation of cysteine residues during crystallization has been reported in other crystal structures (33, 34). The active site, which is located between the two domains, has the glutathionyl moiety (GS moiety) of the GS-AV substrate sitting on the top of the four β-strands of the N-terminal thioredoxin domain and with the acetoveratrone moiety (AV moiety) of the substrate contacting residues from α4, α8, and a cap loop region, ^220^GGGNG^224^, from the C-terminal α-helical domain (Fig. 4*d*). The catalytic residue Cys^15^, located in the loop between β1 and α1, was observed in two distinct conformations with distances of 3.6 and 4.7 Å from the cysteine sulfur atom to the sulfur atom of GS-AV. The glutathionyl moiety of the substrate contacts the loops of the thioredoxin-like domain β1/α1 (residues Ile^12^, Cys^15^, and Phe^17^), α2/β3 (residues Thr^55^, Ala^56^, Leu^57^, and Pro^58^), and β4/α3 (residues Glu^70^ and Ser^71^). The AV moiety of the substrate contacts the C-terminal α-helical domain via residues Ser^109^, Tyr^113^, and Leu^117^ located in α4 and the residues Tyr^217^ and Asn^223^ located in α8 and the cap loop, respectively (Fig. 4*d*). The residue Tyr^113^ in α4 interacts via π-π stacking with the aromatic ring of the substrate (Fig. 4*d*). A break in the electron density was observed between the GS moiety and the AV moiety of the GS-AV analog substrate (Fig. 4*c*) in the feature-enhanced map (35). We postulate that this is a result of the paucity of atomic contacts between the LigG and GS-AV in this region, allowing increased flexibility of the ligand.

##### Lyase Enzymatic Analysis

The thiol transferase activity of LigG was probed with two substrates, GS-AV (supplemental Fig. 1) and (β*R*)-GS-HPV (Fig. 1), at pH 9.0. As reported previously, LigG had little to no activity with (β*S*)-GS-HPV, and the mutation of Cys^15^ to serine abolished enzyme activity with (β*R*)-GS-HPV (30). The *K_m_* measured for GS-AV was ∼12-fold higher than for (β*R*)-GS-HPV, whereas the *k*_cat_ was unchanged (Table 3). These results suggest that the active site of LigG has evolved to tightly bind the GS-conjugated product from LigF, (β*R*)-GS-HPV.

**TABLE 3:** **Kinetic parameters, determined from Michaelis-Menten curves for GSH-dependent glutathione lyase LigG with substrates GS-AV and (β*R*)-GS-HPV at pH 9.0**

Enzyme	Substrate	*V*_max_	Percentage of WT activity with (β*R*)-GS-HPV	*k*_cat_	*K_m_*	*k*_cat_/*K_m_*
		*units mg*^−*1*^	%	*s*^−*1*^	μ*m*	*mm*^−*1*^ *s*^−*1*^
LigG	GS-AV	83.7 ± 7.1	105	29.1 ± 1.4	204.6 ± 16.9	142 ± 14
LigG	(β*R*)-GS-HPV	79.5 ± 9.1	100	27.7 ± 1.2	16.2 ± 1.4	1710 ± 169
LigG-I12A	(β*R*)-GS-HPV	30.3 ± 3.5	38			
LigG-I12V	(β*R*)-GS-HPV	58.8 ± 6.3	74			
LigG-N223A	(β*R*)-GS-HPV	17.8 ± 1.7	22			
LigG-N223G	(β*R*)-GS-HPV	31.6 ± 3.4	40			
LigG-N223V	(β*R*)-GS-HPV	2.3 ± 0.2	3			
LigG-Y217F	(β*R*)-GS-HPV	0.1 ± 0.01	<1			
LigG-Y113F	(β*R*)-GS-HPV	0.3 ± 0.03	<1			
LigG-Y214F	(β*R*)-GS-HPV	0.1 ± 0.01	<1			
LigG-C15S	(β*R*)-GS-HPV	<0.01	<0.1			

Inspection of the LigG·GS-AV crystal structure shows that residues Ile^12^, Tyr^113^, Tyr^214^, Tyr^217^, and Asn^223^ are able to interact with the GS-AV substrate via van der Waals contacts in either the first or second coordination shell. We investigated the contribution of these residues to LigG activity and stereoselectivity by testing the activity with a series of variant enzymes that contain single amino acid substitutions (Table 3). None of the variants displayed activity toward (β*S*)-GS-HPV, and all had compromised activity with (β*R*)-GS-HPV relative to the wild-type enzyme. We also found that substitution of Tyr^113^, Tyr^214^, or Tyr^217^ by phenylalanine abolished enzyme activity, suggesting that the phenolic hydroxyl groups on these three residues play important roles in substrate binding and/or active site organization (Fig. 4*d*). These tyrosine phenolic groups could additionally be implicated in defining the p*K_a_* of the LigG active site.

## Discussion

With the potential impacts of global climate change and the eventual limit on the availability of fossil fuels, there has been significant emphasis on the development of biological routes to renewable transportation fuels (1). However, it is clear that full economic efficiency will not be realized until the lignin component of biomass can be converted into fuels or other bioproducts (2, 3). In addition, many compounds that are currently derived from fossil fuels could instead be obtained from lignocellulosic biomass, with applications in the chemical, food, and pharmaceutical industries (2). The controlled biochemical conversion of lignin breakdown products to well defined chemical units, such as monomers, has the potential to improve the cost effectiveness of renewable biofuels and further reduce our dependence on fossil fuel for chemicals.

The catabolic pathway for the breakdown of β-aryl ether linkages in lignin from the bacterium *Sphingobium* sp. strain SYK-6 differs fundamentally from fungal pathways in that it is independent of chemical mediators, performs chemistry at specific locations between monomeric units, and makes use of pyridine nucleotides and glutathione as cofactors. The Lig pathway enzymes have also been shown to be active against guaiacyl- and syringyl-containing compounds, suggesting that they are active with lignin-derived materials containing the major aromatic units in plant cell walls (36). Our structural and biochemical studies of the enzymes in the bacterial β-aryl ether cleavage pathway reveal the features important for substrate and cofactor binding and catalysis by these Lig enzymes.

The Cα-dehydrogenase crystal structures show that the catalytic mechanisms of LigD, LigO, and LigL are similar to those of other SDR superfamily member enzymes, in which Cα-oxidation of GGE commences with base-catalyzed deprotonation of the Cα-hydroxyl group, followed by ketone formation coupled with hydride transfer from Cα to NAD^+^, resulting in the coproducts NADH and MPHPV. Similarly with other SDRs, basic conditions were optimal for the Cα-dehydrogenation reactions, indicating that Cα-hydroxyl deprotonation is a rate-limiting step during catalysis. We also observed that single point mutations in LigL were able to increase the enzymatic activity, suggesting that protein engineering approaches could be used to modify pathway performance.

Our analysis of LigG is consistent with those of other Omega-class GST member enzymes (30). The loss of enzyme activity in the LigG-C15S variant is consistent with the role of the Cys^15^ thiol in the LigG catalytic cycle, in which an initial disulfide bond is formed between the Cys^15^ side chain and the glutathionyl moiety of GS-HPV, thereby releasing HPV. Subsequently, the deprotonated GSH molecule is used to cleave the GS-enzyme disulfide linkage, in an exchange reaction, yielding GSSG and restored LigG.

Our structural and biochemical studies provide a detailed mechanistic understanding of key components of a bacterial pathway capable of converting lignin breakdown products into defined monomers. When coupled with parallel studies of the Lig etherases and the genetic analysis of this pathway, this knowledge can enable the future optimization of the pathway for the production of specific lignin-derived compounds.

## Author Contributions

J. H. P. designed experiments; solved and analyzed the LigD, LigO, LigL, and LigG structures; co-wrote the initial draft; and edited the manuscript. D. L. G. designed experiments, synthesized substrates, cloned genes, expressed protein, performed enzymatic assays, co-wrote the initial draft, and edited the manuscript. R. A. H. designed experiments, cloned genes, expressed protein, performed enzymatic assays, and edited the manuscript. R. P. M. performed crystallographic data collection of LigG. K. D. synthesized substrates. K. C. H. produced protein. T. J. D. provided research direction, contributed microbiological specific expertise, and edited the manuscript. D. R. N. designed experiments, led enzymology on LigG, provided substrates, and edited the manuscript. B. A. S. provided research direction, contributed lignin-specific and ligninolytic enzyme expertise, and edited the manuscript. K. L. S. provided research direction, contributed lignin-specific and ligninolytic enzyme expertise, and edited the manuscript. J. R. provided research direction, contributed lignin-specific expertise, and edited the manuscript. P. D. A. provided research direction, contributed crystallographic and structural biology expertise, and edited the manuscript.

## Supplementary Material

Supplemental Data

## Footnotes

3 The abbreviations used are:

β-ether: β-aryl ether
GGE: guaiacylglycerol-β-guaiacyl ether
MPHPV: β-(2-methoxyphenoxy)-γ-hydroxypropiovanillone
GS-HPV: β-glutathionyl
HPV: γ-hydroxypropiovanillone
AV: acetoveratrone
Bistris propane: 1,3-bis[tris(hydroxymethyl)methylamino]propane
SDR: short chain dehydrogenase/reductase.
