# Supplementary material for: Structural and Biochemical Characterization of the Early and Late Enzymes in the Lignin β-Aryl Ether Cleavage Pathway from Sphingobium sp. SYK-6
Source: J Biol Chem. 2016 Mar 3;291(19):10228–38. doi: 10.1074/jbc.M115.700427 (PMC4858972; doi:10.1074/jbc.M115.700427)
Supplement: Supplemental Data [file supp_291_19_10228__index.html]

Structural and Biochemical Characterization of the Early and Late Enzymes in the Lignin β-aryl Ether Cleavage Pathway from Sphingobium sp SYK-6 — Structural and Biochemical Characterization of the Early and Late Enzymes in the Lignin β-Aryl Ether Cleavage Pathway from Sphingobium sp. SYK-6 — Structural Studies of Lignin β-Ether-cleaving Enzymes — Supplemental Data 

# Structural and Biochemical Characterization of the Early and Late Enzymes in the Lignin β-Aryl Ether Cleavage Pathway from *Sphingobium* sp. SYK-6

## Supplemental Data

- Supporting Information (.pdf, 2.1 MB) - Supporting Information File
