# Supplementary material for: Structural and Biochemical Characterization of the Early and Late Enzymes in the Lignin β-Aryl Ether Cleavage Pathway from Sphingobium sp. SYK-6
Source: J Biol Chem. 2016 Mar 3;291(19):10228–38. doi: 10.1074/jbc.M115.700427 (PMC4858972; doi:10.1074/jbc.M115.700427)
Supplement: Supplemental Data [file 10.1074_M115.700427_jbc.M115.700427-1.pdf]

SUPPORTING INFORMATION

Structural and biochemical characterization of the early and late enzymes in the lignin β-aryl ether cleavage pathway from *Sphingobium* sp SYK-6

Jose Henrique Pereira[1,2#], Richard A. Heins[1,3,#], Daniel L. Gall[4,5,#], Ryan P. McAndrew[1,2], Kai Deng[1,3], Keefe C. Holland[1,3], Timothy J. Donohue[4,6], Daniel R. Noguera[4,5], Blake A. Simmons[1,3], Kenneth L. Sale[1,3], John Ralph[4,6], Paul D. Adams[1,2,7]

[1]Joint BioEnergy Institute, Emeryville, CA 94608, USA, [2]Molecular Biophysics and Integrated Bioimaging Division, Lawrence Berkeley National Laboratory, Berkeley, CA 94720, USA, [3]Biological and Engineering Sciences Center, Sandia National Laboratories, Livermore, CA, USA 94551 USA, [4]U.S. Department of Energy Great Lakes Bioenergy Research Center, Wisconsin Energy Institute, University of Wisconsin, Madison, WI, 53726, [5] Department of Civil & Environmental Engineering, University of Wisconsin, Madison, WI, 53706, [6]Department of Biochemistry, University of Wisconsin, Madison, WI 53706, [7]Department of Bioengineering, University of California Berkeley, CA 94720, USA, [#]These authors contributed equally to this work.

Running title: Structural studies of β-aryl ether bond cleaving enzymes

To whom correspondence should be addressed: Paul D. Adams (*pdadams@lbl.gov*)

Table of contents

	Page
**Supporting Figure 1 -** Structure of the GS-HPV	**S-3**
**Supporting Figure 2 -** Gene construct sequence for pCPD-LigL	**S-4**
**Supporting Figure 3 -** Sequence alignment between the Cα dehydrogenases	**S-8**
**Supporting Figure 4 -** Electron density maps for the Cα dehydrogenases	**S-10**
**Supporting Figure 5** – Molecular model of LigL-NADH-(α*S*,β*S*)-GGE	**S-11**
**Supporting Figure 6 -** The dehydrogenation of GGE to MPHPV	**S-13**
**Supporting Figure 7** – Oxidation of the cysteines	**S-14**

Supporting Figure 1

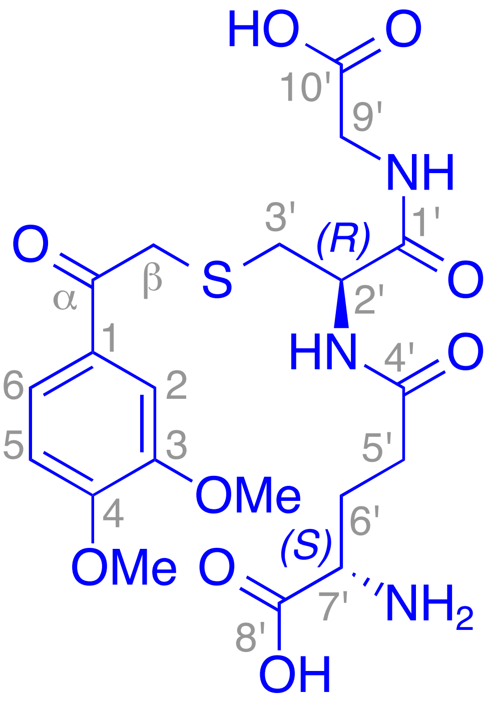

GS-AV

Supporting Figure 1 - a) Structure of the GS-HPV analog substrate, GS-AV, which was used in the crystallization of LigG.

Supporting Figure 2

ggttattgtctcatgagcggatacatatttgaatgtatttagaaaaataaacaaataggggttccgcgca
catttccccgaaaagtgccacctgacgtctaagaaaccattattatcatgacattaacctataaaaatag
gcgtatcacgaggccctttcgtcttcacctcgagaaatcataaaaaatttatttgctttgtgagcggata
acaattataatagattcaattgtgagcggataacaatttcacacagaattcattaaagaggagaaattaa
cgATGGACATCGCAGGCACAACAGCGTTCATCACTGGCGGCGCATCGGGCATCGGTTTCGGTATCGCACA
AAGATTACTGGCAAATGGTGCTCGCTTGGTTCTGGCAGATATTCGTCAAGATCATCTGGACGAAGCCCGT
CAGTTTTTCGAAGAACGCCAACAGGGTCGTAATGTCCACACTATTAGATTAGATGTATCTGACCGCGCCC
AAATGGCGGAAGCTGCAAGAGAATGTGAAGCCGTGATGGGTGGCCCAGATATTCTGATCAATAACGCGGG
TATTGATCCGTCAGGCCCTTTTAAAGACGCTACTTATCAGGATTGGGACTACGGTCTGGCAATCAATTTA
ATGGGTCCAATTAACGGCATCATGGCCTTCACACCTGGTATGCGTGCGAGAGGCCGCGGTGGCCATATTG
TCAACACGGCTAGCTTAGCAGGCTTGACCCCAATGCCGAGTTTTATGGCTATCTATGCTACGGCAAAAGC
CGCGGTTATTACCTTAACTGAAACAATCCGCGATAGTATGGCAGAAGACAATATTGGTGTCACCGTATTG
ATGCCTGGCCCAATTAAGTCTCGTATTCATGAATCAGGTCAAAACCGCCCTGAACGTTTCAGAGCCGGTT
CTGGCTTGGCGGAAACGGAACAACAGCTGGCTAAACGTGTTGTGGCAGATAATTGGATGGAACCAACCGA
AGTTGGTGATATGATTGTTGACGCTATCGTGCATAATAAGCTGTACGTGTCCACTCACGGCAACTGGCGT
GAAACATGCGAAGCCAGATTTCAGGCGCTGTTAGATTCGATGCCTGAAGCAAGACCGTTTGATTTTGGTG
CCTCGCTGGCTGTGCCTAAAGAAGAAGCActcgcagatggtaaaattctgcataatcagaatgtgaatag
ctggggtccgattaccgttaccccgaccaccgatggtggtgaaacccgttttgatggtcagattattgtg
cagatggaaaatgatccggttgttgcaaaagcagcagcaaatctggcaggtaaacatgcagaaagcagcg
ttgttgttcagctggatagtgatggtaattatcgtgttgtttatggcgatccgagcaaactggatggtaa
actgcgttggcagctggttggtcatggtcgtgatcatagcgaaaccaataatacccgtctgagcggttat
agcgcagatgaactggcagttaaactggcaaaatttcagcagagctttaatcaggccgaaaacattaata
acaaaccggatcatatttcgattgtgggttgtagcctggtgagtgatgataaacagaaaggttttggcca
ccagtttattaacgcaatggatgcaaatggtctgcgtgttgatgttagcgttcgtagcagcgaactggcc
gttgatgaagcaggtcgtaaacacaccaaagatgccaatggtgattgggttcagaaagccgaaaataaca
aagttagc**ATT**agctgggatgcacagggcggttctggtgagaat**GCA**tactttcaaggatggtcacatcc
acaattcgaaaagaatagcgccgtcgacggtagcggaCATCATCATCATCATCATCATCATtgaggcagt
tattggtgcccttaaacgcctggggtaatgactctctagcttgaggcatcaaataaaacgaaaggctcag
tcgaaagactgggcctttcgttttatctgttgtttgtcggtgaacgctctcctgagtaggacaaatccgc
cctctagattacgtgcagtcgatgataagctgtcaaacatgagaattgtgcctaatgagtgagctaactt
acattaattgcgttgcgctcactgcccgctttccagtcgggaaacctgtcgtgccagctgcattaatgaa

tcggccaacgcgcggggagaggcggtttgcgtattgggcgccagggtggtttttcttttcaccagtgaga
cgggcaacagctgattgcccttcaccgcctggccctgagagagttgcagcaagcggtccacgctggtttg
ccccagcaggcgaaaatcctgtttgatggtggttaacggcgggatataacatgagctgtcttcggtatcg
tcgtatcccactaccgagatatccgcaccaacgcgcagcccggactcggtaatggcgcgcattgcgccca
gcgccatctgatcgttggcaaccagcatcgcagtgggaacgatgccctcattcagcatttgcatggtttg
ttgaaaaccggacatggcactccagtcgccttcccgttccgctatcggctgaatttgattgcgagtgaga
tatttatgccagccagccagacgcagacgcgccgagacagaacttaatgggcccgctaacagcgcgattt
gctggtgacccaatgcgaccagatgctccacgcccagtcgcgtaccgtcttcatgggagaaaataatact
gttgatgggtgtctggtcagagacatcaagaaataacgccggaacattagtgcaggcagcttccacagca
atggcatcctggtcatccagcggatagttaatgatcagcccactgacgcgttgcgcgagaagattgtgca
ccgccgctttacaggcttcgacgccgcttcgttctaccatcgacaccaccacgctggcacccagttgatc
ggcgcgagatttaatcgccgcgacaatttgcgacggcgcgtgcagggccagactggaggtggcaacgcca
atcagcaacgactgtttgcccgccagttgttgtgccacgcggttgggaatgtaattcagctccgccatcg
ccgcttccactttttcccgcgttttcgcagaaacgtggctggcctggttcaccacgcgggaaacggtctg
ataagagacaccggcatactctgcgacatcgtataacgttactggtttcacattcaccaccctgaattga
ctctcttccgggcgctatcatgccataccgcgaaaggttttgcaccattcgatggtgtcggaatttcggg
cagcgttgggtcctggccacgggtgcgcatgatctagagctgcctcgcgcgtttcggtgatgacggtgaa
aacctctgacacatgcagctcccggagacggtcacagcttgtctgtaagcggatgccgggagcagacaag
cccgtcagggcgcgtcagcgggtgttggcgggtgtcggggcgcagccatgacccagtcacgtagcgatag
cggagtgtatactggcttaactatgcggcatcagagcagattgtactgagagtgcaccatatgcggtgtg
aaataccgcacagatgcgtaaggagaaaataccgcatcaggcgctcttccgcttcctcgctcactgactc
gctgcgctcggtcgttcggctgcggcgagcggtatcagctcactcaaaggcggtaatacggttatccaca
gaatcaggggataacgcaggaaagaacatgtgagcaaaaggccagcaaaaggccaggaaccgtaaaaagg
ccgcgttgctggcgtttttccataggctccgcccccctgacgagcatcacaaaaatcgacgctcaagtca
gaggtggcgaaacccgacaggactataaagataccaggcgtttccccctggaagctccctcgtgcgctct
cctgttccgaccctgccgcttaccggatacctgtccgcctttctcccttcgggaagcgtggcgctttctc
atagctcacgctgtaggtatctcagttcggtgtaggtcgttcgctccaagctgggctgtgtgcacgaacc
ccccgttcagcccgaccgctgcgccttatccggtaactatcgtcttgagtccaacccggtaagacacgac
ttatcgccactggcagcagccactggtaacaggattagcagagcgaggtatgtaggcggtgctacagagt
tcttgaagtggtggcctaactacggctacactagaaggacagtatttggtatctgcgctctgctgaagcc
agttaccttcggaaaaagagttggtagctcttgatccggcaaacaaaccaccgctggtagcggtggtttt
tttgtttgcaagcagcagattacgcgcagaaaaaaaggatctcaagaagatcctttgatcttttctacgg
ggtctgacgctcagtggaacgaaaactcacgttaagggattttggtcatgagattatcaaaaaggatctt

cacctagatccttttaaattaaaaatgaagttttaaatcaatctaaagtatatatgagtaaacttggtct
gacagttagaaaaactcatcgagcatcaaatgaaactgcaatttattcatatcaggattatcaataccat
atttttgaaaaagccgtttctgtaatgaaggagaaaactcaccgaggcagttccataggatggcaagatc
ctggtatcggtctgcgattccgactcgtccaacatcaatacaacctattaatttcccctcgtcaaaaata
aggttatcaagtgagaaatcaccatgagtgacgactgaatccggtgagaatggcaaaagtttatgcattt
ctttccagacttgttcaacaggccagccattacgctcgtcatcaaaatcactcgcatcaaccaaaccgtt
attcattcgtgattgcgcctgagcgagacgaaatacgcgatcgctgttaaaaggacaattacaaacagga
atcgaatgcaaccggcgcaggaacactgccagcgcatcaacaatattttcacctgaatcaggatattctt
ctaatacctggaatgctgttttcccggggatcgcagtggtgagtaaccatgcatcatcaggagtacggat
aaaatgcttgatggtcggaagaggcataaattccgtcagccagtttagtctgaccatctcatctgtaaca
tcattggcaacgctacctttgccatgtttcagaaacaactctggcgcatcgggcttcccatacaatcgat
agattgtcgcacctgattgcccgacattatcgcgagcccatttatacccatataaatcagcatccatgtt
ggaatttaatcgcggcctagagcaagacgtttcccgttgaatatggctcatactcttcctttttcaatat
tattgaagcatttatcag

Supporting Figure 2 - Gene construct sequence for pCPD-LigL that makes use of the *Vibro cholera* MARTX toxin cysteine protease domain (CPD). LigD, LigO and LigG were cloned into this same vector in place of the LigL gene. The original plasmid was constructed such that a leucine was placed in between the protein of interest and CPD; upon activation by InsP6, CPD cleaves the peptide bond after this leucine. CPD is known to be promiscuous and, during protein purification, it was found that the protease cleaved at two additional leucine residues at the C-terminal end of the protein; L203 (numbering refers to the truncated CPD gene) and a leucine inside of a TEV cleavage site in the flexible spacer. Both leucines were mutated; L203 to isoleucine and the leucine in the TEV site to an alanine. These mutations eliminated the observed self-cleavage.

Color legend:

- Green: LigL gene
- Yellow: Leucine cleavage site introduced in between LigL gene and CPD module
- Cyan: CPD (L203I is indicated in bold, underlined, capitalized text)

- Magenta: Flexible linker comprised of a mutated TEV cleavage site and a *StreptII* tag (the leucine to alanine mutation is indicated with capitalized bold text)
- 10X his tag indicated by underlined, capitalized text
- Red: Stop codon

Supporting Figure 3

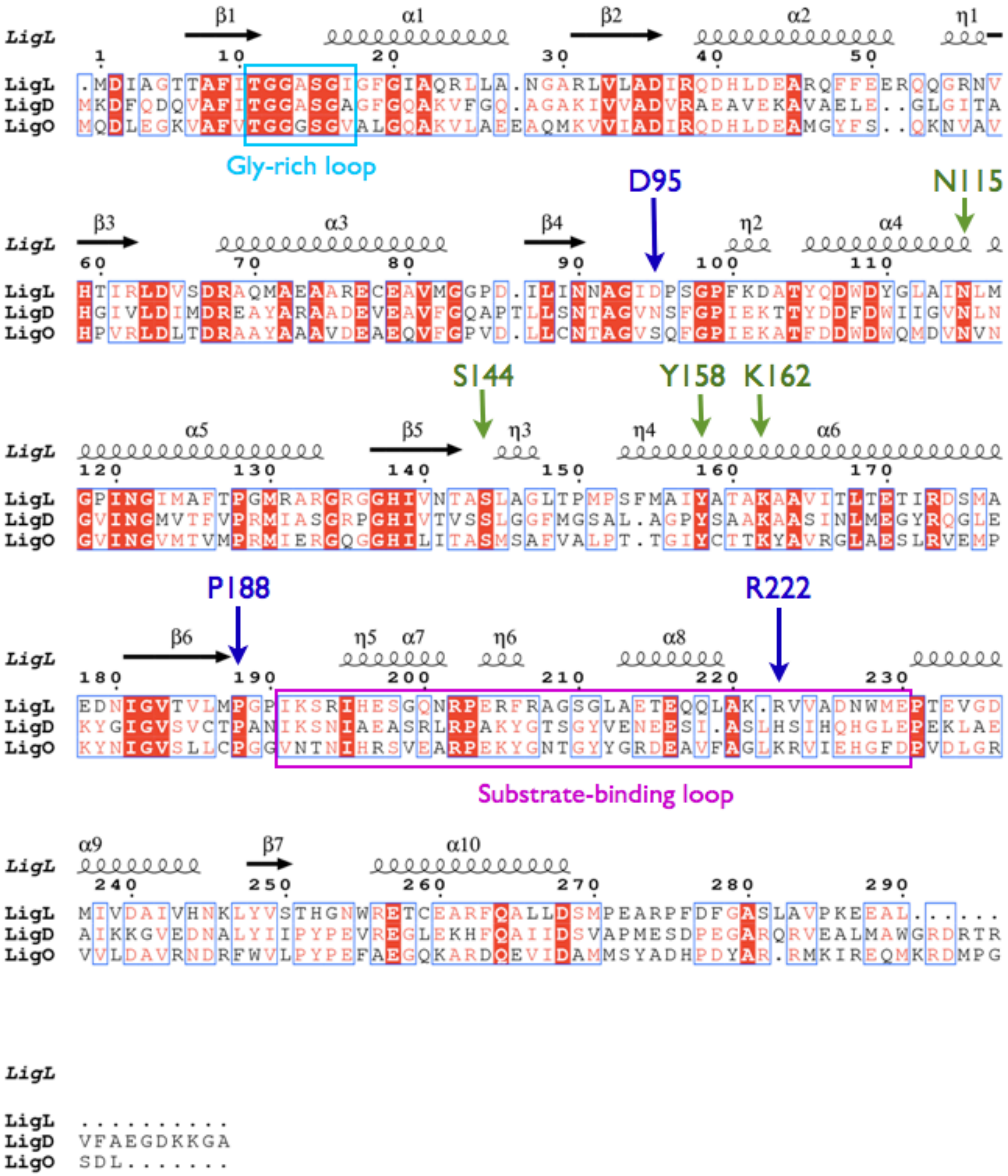

Supporting Figure 3 - The sequence alignment between the Cα dehydrogenases LigD, LigO and LigL shows an identity between LigD-LigO, LigD-LigL and LigO-LigL of 40%, 38% and 37% respectively. The Glycine-rich loop consensus sequences (cyan box) located at N-terminal region ${}_{11}$TGXXXGXG/A${}_{18}$ observed in all SDR family members are in contact with pyro-

phosphate group of NADH cosubstrate. Green arrows indicate the catalytic tetrad of SDR members N115-S144-Y158-K162. Blue arrows indicate the residues D-95, P-188 and R-222 of LigL that make direct contacts with GGE substrate. The most sequence-divergent region is the substrate-binding loop shown inside the magenta box.

Supporting Figure 4

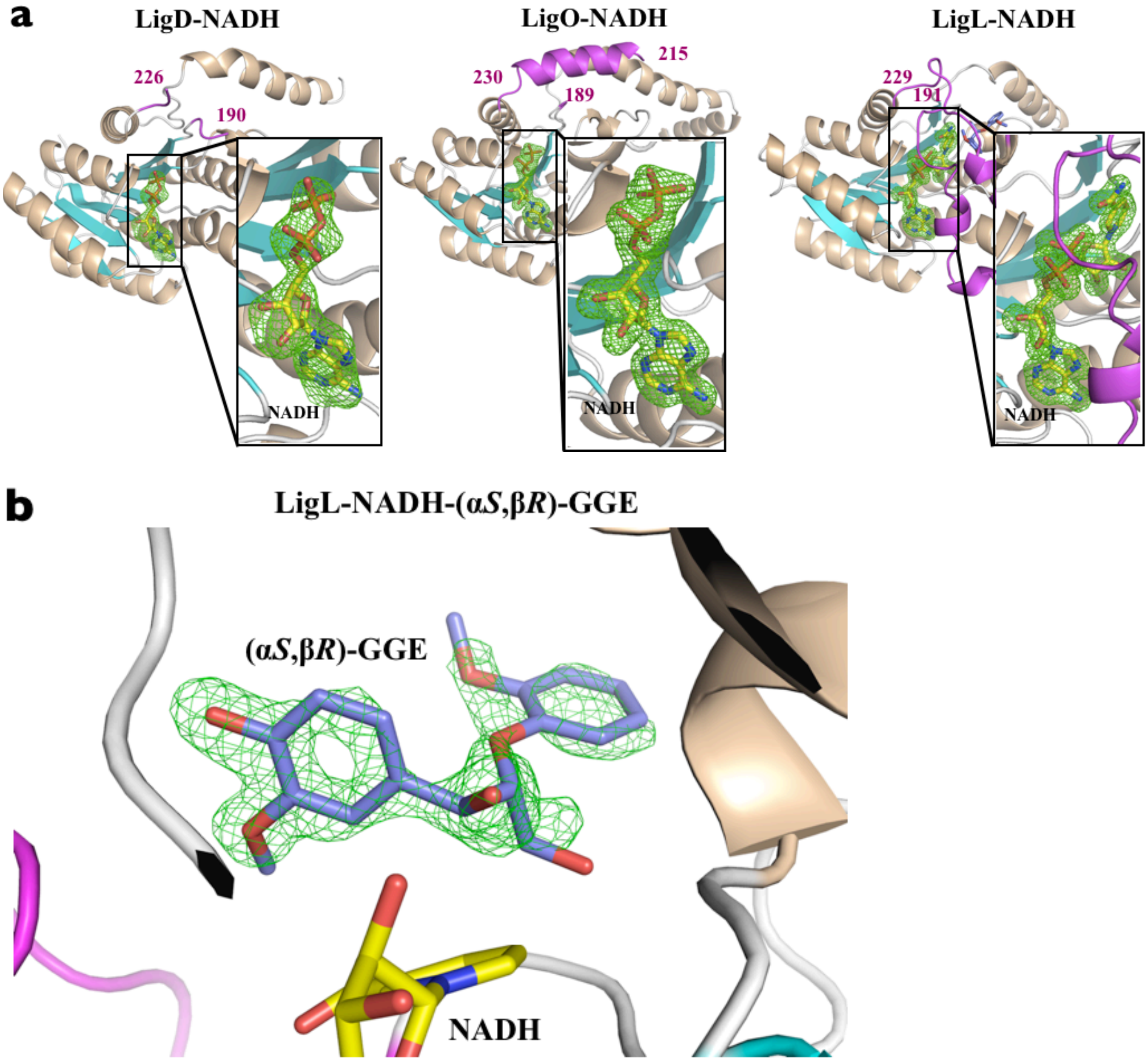

Supporting Figure 4 - a) A mF_o-DF_c electron density map omitting the NADH nucleotide molecule contoured at 3.0 σ is shown in green. The adenine part of cosubstrate was defined on the electron density maps whereas the nicotinamide portion is missing from LigD-NADH and LigO-NADH structures. **b)** The ternary complex structure of LigL-NADH-(α*S*,β*R*)-GGE show a clear mF_o-DF_c electron density map contoured at 2.8 σ in green omitting the (α*S*,β*R*)-GGE substrate.

Supporting Figure 5

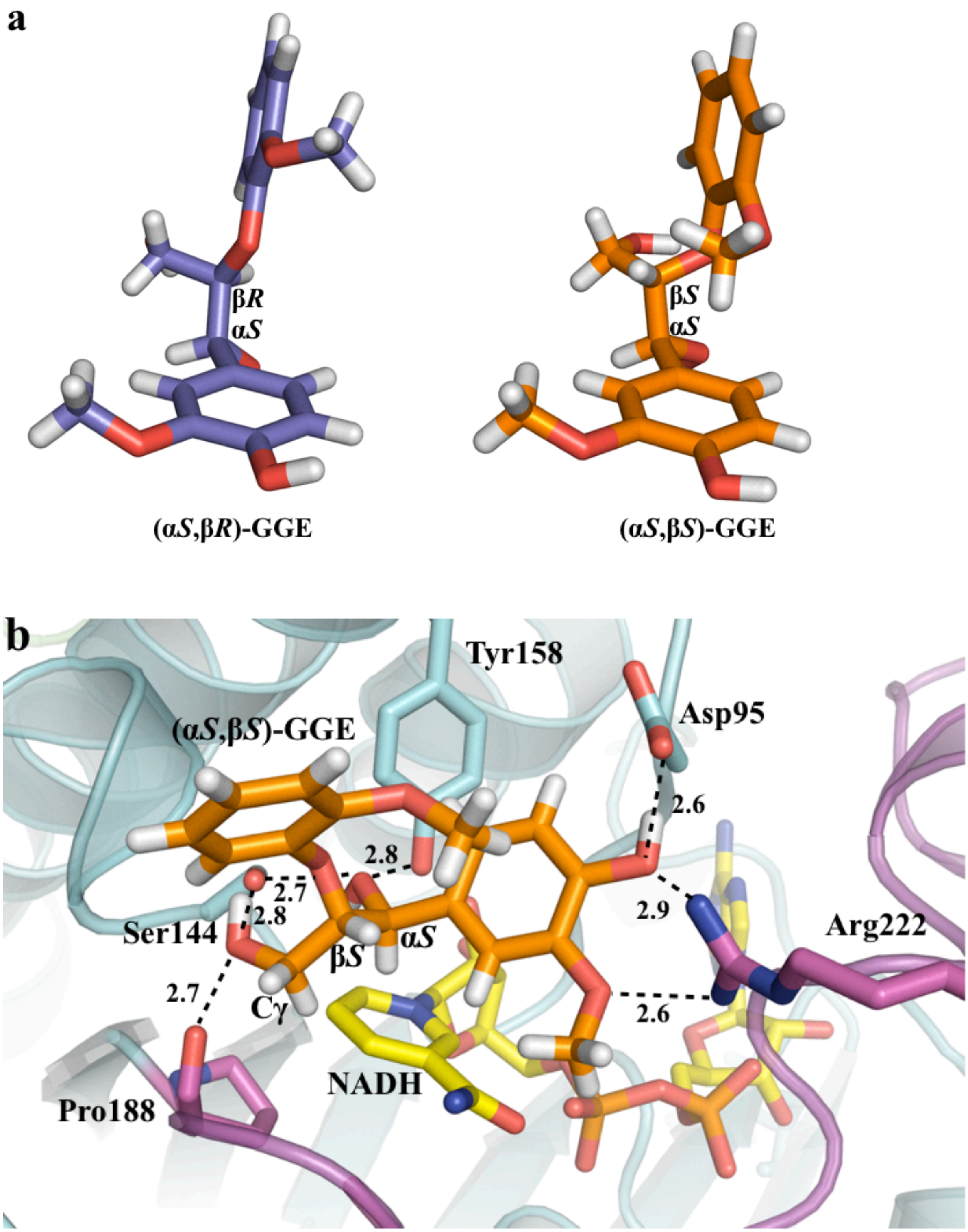

Supporting Figure 5 - a) Schematic representation of α(*S*)-configured substrates (α*S*,β*R*)-GGE and (α*S*,β*S*)-GGE showing the two alternative chiral centers, *R* and *S,* present at the Cβ of the GGE molecule. **b)** Substrate binding site for the molecular model of the LigL-NADH-(α*S*,β*S*)-GGE complex showing residues Asp95, Ser144, Tyr158, Pro188 and Arg222 that interact directly with the GGE substrate. An extra hydrogen bond between Ser144 and a hydroxyl group attached to Cγ of (α*S*,β*S*)-GGE is observed in this model compared to the crystal structure of LigL-NADH-(α*S*,β*R*)-GGE. Broken lines represent hydrogen bonds and distances are shown in Angstroms.

Supporting Figure 6

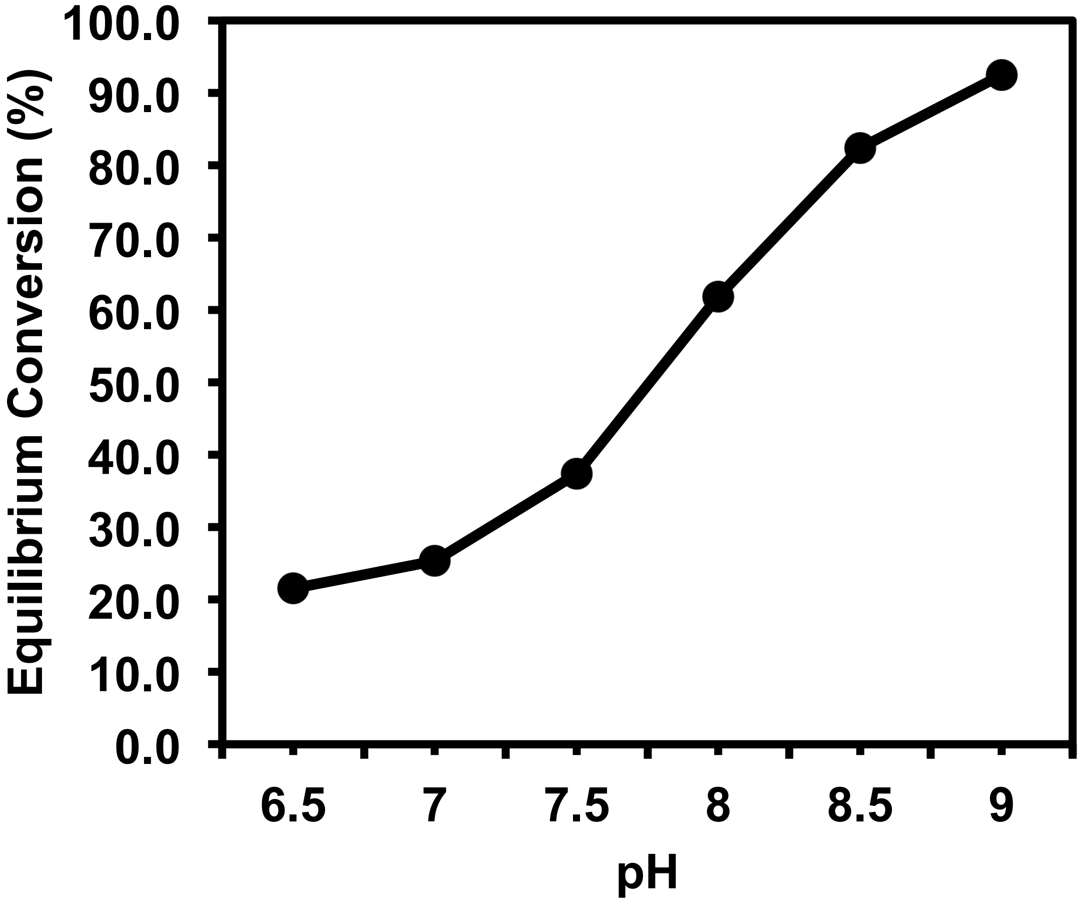

Supporting Figure 6 - The dehydrogenation of GGE to MPHPV by LigL is strongly governed by solution pH, with equilibrium conversion reaching 90% at pH 9 but only 25% at pH 7. A phosphate buffer was used for pH 6.5 through 7.5, and a Tris buffer was used for pH 8 through 9.

Supporting Figure 7

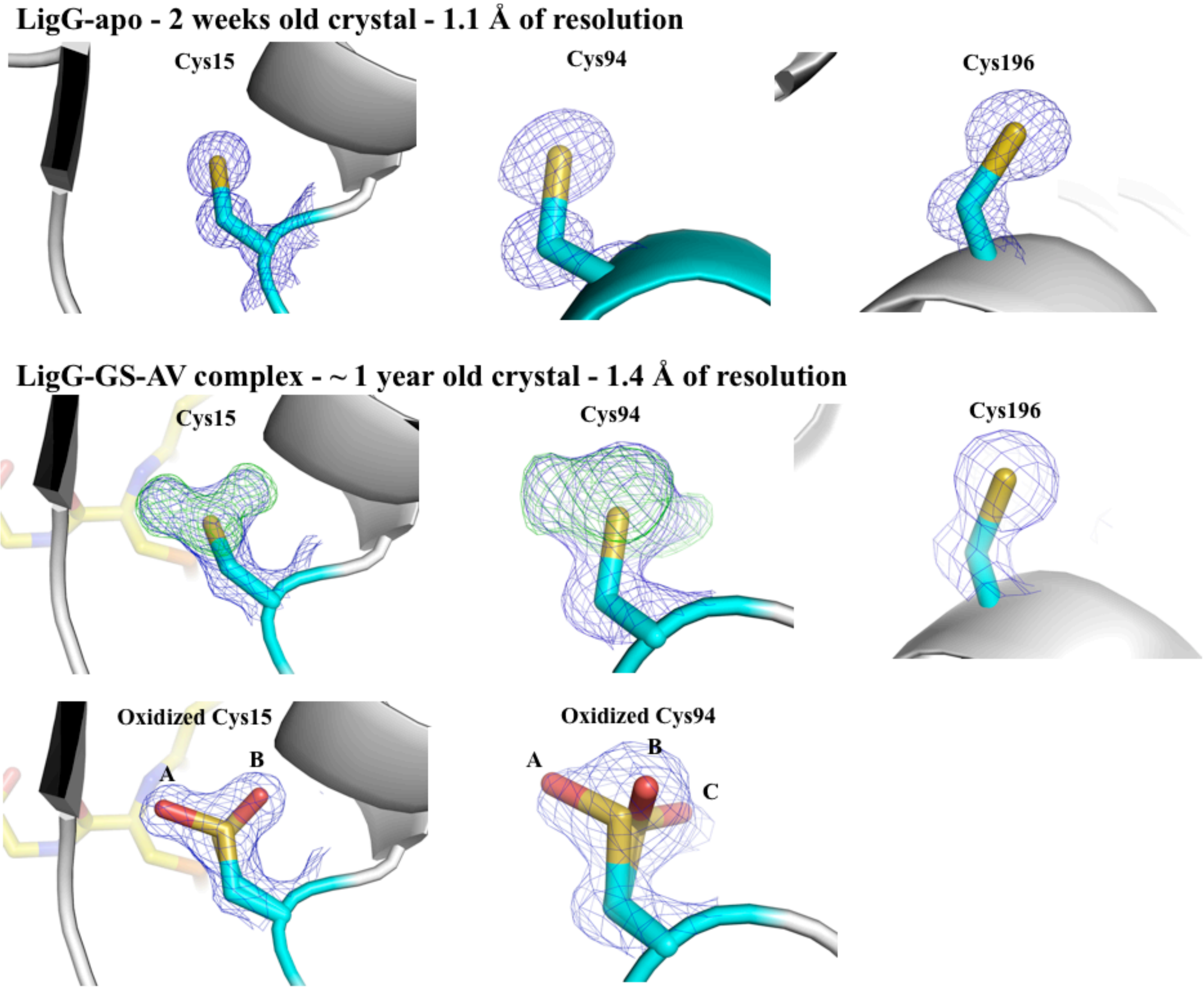

Supporting Figure 7 - A LigG-apo $2mF_o$-DF_c electron density map contoured at 1.0 σ (blue) from a 2-weeks old crystal shows no additional density around the sulfur atoms of Cys15 (catalytic residue), Cys94 and Cys196. Approximately 1-year old crystals of apo-LigG were used for soaking experiments using GS-AV ligand. These older crystals showed clear additional mF_o-DF_c electron density in maps contoured at 3.0 σ (green) around the sulfur atoms of Cys15 and Cys94 indicating the oxidation of these residues, but not for Cys196. Two alternative conformation (A and B) for oxidized Cys15 and three

alternative conformations (A, B and C) for oxidized Cys94 were modeled in the final LigG-GS-AV complex, and are shown with the final $2mF_o$-DF_c electron density map contoured at 1.0 σ (blue).
